# Evaluation of patient outcomes after operative treatment of intra-articular calcaneus fractures

**DOI:** 10.1051/sicotj/2021065

**Published:** 2021-12-31

**Authors:** Kevin Steelman, Nicholas Bolz, Enrique Feria-Arias, Robert Meehan

**Affiliations:** Detroit Medical Center, Department of Orthopaedic Surgery, Harper University Hospital 3990 John R Detroit MI 48201 USA

**Keywords:** Calcaneus fractures, Closed reduction percutaneous fixation, Open reduction internal fixation

## Abstract

*Background*: Percutaneous reduction with fixation and open reduction internal fixation are often used to treat intra-articular calcaneus fractures with no consensus on the preferred method. Open techniques have been associated with an increased risk of wound complications, while percutaneous techniques may result in inferior reduction capabilities. These injuries pose a challenge to patients as they often result in poor patient outcomes. We retrospectively analyzed patient outcomes of a single surgeon’s experience in treating these injuries at a busy urban Level 1 trauma center. *Methods*: Patients with intra-articular calcaneus fractures managed operatively over 10 years with a minimum six-month follow-up were included. Patients were divided into two cohorts based on operative technique: closed reduction and percutaneous fixation (CRPF) or open reduction internal fixation (ORIF). Descriptive analysis of each cohort included postoperative infection, the need for repeat operations, development of post-traumatic subtalar arthritis, and reduction capabilities as assessed by Bohler’s angle. *Results*: Sixty-two patients were included in this study, with 33 patients in the CRPF group and 29 patients in the ORIF group. Infection requiring a return to the operating room occurred in 1 (3%) CRPF and 7 (24%) ORIF patients. Instrumentation was removed in 23 (70%) CRPF and 9 (31%) ORIF patients. Clinical subtalar arthritis developed in 10 (30%) CRPF and 7 (24%) ORIF patients, requiring arthrodesis in 2 (6%) and 5 (17%) patients, respectively. Both techniques had acceptable restoration of Bohler’s angle immediately postoperatively and at final follow-up. *Conclusions*: Percutaneous reduction with fixation and open reduction internal fixation may both be considered for the surgical treatment of intra-articular calcaneal fractures. Indications for each technique may vary between surgeons, and each has its own set of risk factors and complications, however, both have been shown to result in an acceptable reduction. *Level of Evidence*: Level IV.

## Introduction

The annual incidence of calcaneus fractures is approximately 11.5 per 100,000, representing nearly 2% of all fractures [1]. Calcaneal fractures can lead to severe long-term pain and disability, making proper management critical. Despite this, there remains a lack of consensus in the literature between operative vs. nonoperative care, as well as operative technique.

The goal of calcaneal fracture treatment, as defined by the American Orthopedic Foot and Ankle Society (AOFAS), is to restore the normal alignment and contour of the calcaneus. This is achieved by restoring heel height and length (frequently determined by the measurement of Bohler’s angle), the realignment of the posterior facet of the subtalar joint, and restoring the mechanical axis of the foot [1].

Current indications for operative treatment include displaced intra-articular posterior facet fractures, anterior process fractures with more than 25% involvement of the calcaneocuboid joint, displaced fractures of the calcaneal tuberosity, fracture-dislocations, and select open fractures [2]. As new operative techniques gain momentum, surgeons have increasing options for intervention. These include but are not limited to: open reduction internal fixation (ORIF) via extended L-shaped incision, limited incision sinus tarsi ORIF, closed reduction with percutaneous fixation (CRPF), and arthroscopic-assisted fixation. Given the increased risk for wound complications with operative treatment, conservative management is often recommended when appropriate [3–9]. Even when calcaneal fracture patterns meet operative indications, the optimal operative technique remains controversial.

This study attempts to add to the literature on an already controversial topic by describing patient outcomes after operative treatment of intra-articular calcaneus fractures in two groups of patients: 1) Those treated with ORIF through an extensive lateral approach, and 2) those treated with CRPF. Outcomes investigated in this study included rates of infection [1–6, 10–13], removal of instrumentation [14, 15], development of post-traumatic subtalar arthritis [3, 16], and reduction capabilities of each method through the restoration and maintenance of Bohler’s angle [2, 7, 10, 17–20]. This study is relevant as patients with these injuries continue to struggle with long-term complications and chronic pain after operative treatment.

## Materials and methods

After obtaining Institutional Review Board approval, we conducted a retrospective review of all intra-articular calcaneal fractures treated over 10 years (October 2003 to September 2013) at a large, urban, level 1 trauma center. Patients were identified using the electronic medical record (EMR). All procedures were performed by a single foot and ankle fellowship-trained orthopedic surgeon with significant experience utilizing both techniques. An extensile lateral approach was used in the ORIF cohort, and a combination of percutaneous screws was used in the CRPF cohort after restoring calcaneal anatomy through the use of a universal distractor and Schanz pins to joystick fragments into anatomic position.

The decision to proceed with CRPF or ORIF was dictated by the following protocols as dictated by the senior author:

- CRPF: Simple, two-part facet patterns less than 1–2 weeks old, or in patients with complex medical comorbidities or social situations such as homelessness. Also, bilateral injuries, regardless of fracture pattern if they were able to be operated on within 7–10 days.
- ORIF: Fracture patterns involving more than two pieces of the posterior facet, significant comminution of the facet, or was older than 2–3 weeks old.

Inclusion criteria required patients over the age of 18, fractures involving the posterior facet, fractures undergoing ORIF using an extensile lateral approach or minimally invasive CRPF, and at least six months of clinical follow-up postoperatively. Minimally invasive CRPF technique was defined as closed reduction maneuvers only, with no open exposure to the subtalar joint. Patients who sustained a complication or reoperation prior to the six-month follow-up were included in the analysis to minimize any positive bias. Patients failing to meet these criteria or without intra-articular fractures were excluded.

Demographic data included age, gender, race, mechanism of injury, open versus closed fracture, the time between injury and surgery, diabetes, tobacco use, and worker’s compensation status. Fractures were classified according to the Sanders Classification on preoperative computed tomography (CT). The classification was performed by one of the authors (EF) and confirmed by the single operative surgeon (RM). Bohler’s angle was measured on lateral X-ray preoperatively, postoperatively, and at the final clinical follow-up.

Clinical outcomes measured included 1) development of infection, 2) removal of instrumentation, 3) development of post-traumatic subtalar arthritis, and 4) postoperative calcaneal reduction as determined by restoration of Bohler’s angle on lateral radiographs. Postoperative infections were considered superficial if they were treated with oral antibiotics without operative intervention. Infections were considered deep if they required IV antibiotics and/or operative intervention (e.g., irrigation and debridement). Development of post-traumatic subtalar arthritis was diagnosed on clinical presentation (symptoms requiring steroid injection or subtalar arthrodesis) rather than radiographic criteria. Bohler’s angle was measured at three distinctive time periods in each group: preoperatively, immediately postoperatively, and at final follow-up.

Descriptive statistics were used to evaluate significance using an alpha factor of 0.05. Categorical variables were evaluated with chi-square testing analysis, while continuous variables utilized ANOVA analysis. All statistical analysis was performed using IBM SPSS software version 25 (IMB, Armonk, NY, USA).

## Results

A total of 62 fractures in 52 patients were included in this study. Twenty-nine fractures were treated with ORIF, and 33 fractures were treated with CRPF. Patient demographics and mechanism of injury can be viewed in Table 1. All Fractures were classified according to Sander’s classification [21, 22] as well as the Essex-Lopresti classification. Fracture classifications can be viewed in Table 2.

**Table 1:** Patient demographics and mechanism of injury of intra-articular calcaneus fractures treated with open reduction internal fixation (ORIF) and closed reduction percutaneous fixation (CRPF).

Demographic	Study population	CRPF	ORIF
Number of patients (*n*)	62	33	29
Age (mean +/− SD, years)	41.0	43	41
Sex (number [%])			
Males	52	30 (91%)	22 (76%)
Females	10	3 (9%)	7 (24%)
Body Mass Index	26.5	26.6	26.2
Ethnicity (%)			
Caucasian	39	33	45
African American	48	52	45
Hispanic	2	0	3
Native American	2	3	0
Other	10	12	7
Tobacco Use (*n*, [%])	36 (58%)	22 (67%)	14 (48%)
Workers compensation	4 (6%)	2 (6%)	2 (7%)
Open fractures	4 (6%)	2 (6%)	2 (7%)
Mean follow-up (months)	26.0	27.4	23.8
Mean time to surgery (days)*	12	4.5	20.8
Mechanism of injury (*n*)			
Fall	54	29	25
Motor vehicle collision	5	2	3
Motorcycle accident	2	1	1
Assault	0	1	0

**Table 2:** Preoperative Sanders and Essex-Lopresti classification of calcaneus fractures in ORIF and CRPF groups.

Variable	Study population	CRPF	ORIF
Sanders classification			
Type II	42 (68%)	22 (67%)	20 (69%)
IIA	11	4	7
IIB	22	14	8
IIC	9	4	5
Type III	16 (32%)	8 (24%)	8 (31%)
IIIAB	15	8	7
IIIAC	1	0	1
IIIBC	0	0	0
Type IV	2 (3%)	2 (6%)	0 (0%)
Essex-Lopresti classification			
Depression	50 (81%)	25 (76%)	25 (86%)
Tongue-Type	0 (0%)	8 (24%)	4 (14%)

This study found a higher number of infections in the ORIF cohort, and infection was often associated with smoking status and the presence of diabetes in both groups. In the ORIF group, 7 patients (24%) developed a superficial infection, and 7 patients (24%) developed a deep infection. All patients who had a deep infection required reoperation in the form of irrigation and debridement (I&D), with 4/7 (57%) patients requiring more than one I&D (range 2–4). Five of the 7 ORIF deep infections (71%) were smokers, and 2/7 (29%) of the ORIF superficial infections were smokers. Seven out of 14 (50%) ORIF smokers had deep or superficial infections. There were two diabetic patients in the ORIF group (7%), and both had superficial infections (100%). In the CRPF group, four patients (12%) developed a superficial infection, and one patient (3%) developed a deep infection that required I&D. The deep infection patient was a smoker (100%), and three of the four superficial infections (75%) were smokers. Four out of 22 (18%) of CRPF smokers had deep or superficial infections. There were no diabetic patients in the CRPF cohort.

There was also a high number of reoperations in this study, the majority of which were for the removal of instrumentation. Thirty-seven patients (60%) had additional operations within our follow-up interval: 24 (73%) in the CPRF group and 13 (45%) in the ORIF group. Instrumentation was removed in 32 patients, 23 in the CRPF group and 9 in the ORIF group. All postoperative complications and reasons for reoperation can be viewed in Table 3.

**Table 3:** Complications, rates of infection, incidence of post-traumatic osteoarthritis, and reoperation in ORIF and CRPF groups.

Variable	Study population	CRPF	ORIF
Infection	19 (31%)	5 (15%)	14 (48%)
Superficial infection	11 (18%)	4 (12%)	7 (24%)
Deep infection	8 (13%)	1 (3%)	7 (24%)
Subtalar arthritis	17 (21%)	10 (36%)	7 (24%)
Re-operation (number of patients)	37 (60%)	24 (73%)	13 (45%)
Arthrodesis (cases)	7	5	2
I&D (cases)	8	1	7
Hardware removal (cases)	32	23	9

Seven patients (24%) in the ORIF group and 10 patients (36%) in the CRPF group developed symptomatic post-traumatic subtalar arthritis as defined above. Five out of the 7 (71%) patients in the ORIF group with symptomatic subtalar arthritis went on to have a subtalar arthrodesis, as did 2/10 (20%) patients in the CRPF group. Of the seven ORIF patients diagnosed with subtalar arthritis, 3 (43%) underwent a subtalar steroid injection. In the CRPF group, nine of the 10 (90%) patients with symptomatic subtalar arthritis underwent a subtalar steroid injection, with only 1 progressing to a subtalar fusion.

**Figure 1:**
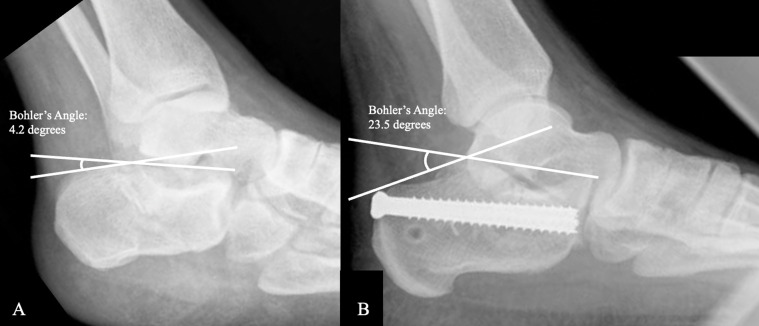
(A) Preoperative lateral foot radiograph of joint depression-type calcaneal fracture. Bohler’s angle measured 4.2 degrees through intersecting lines tangential to the superior tuber and the superior aspect of the posterior facet, and the superior aspect of the posterior facet and the anterior calcaneal process. In (B) a postoperative lateral foot radiograph is shown status post closed reduction with percutaneous fixation with two cannulated, fully threaded 6.5 mm screws with restoration of Bohler’s angle measuring 23.5 degrees.

**Figure 2:**
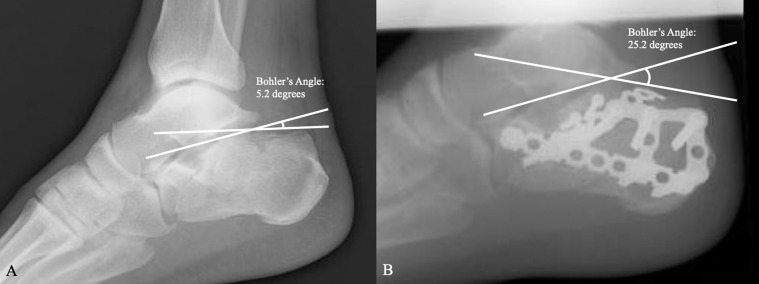
(A) Preoperative lateral foot radiograph of a comminuted, joint depression-type calcaneal fracture. Bohler’s angle measured 5.2 degrees through intersecting lines tangential to the superior tuber and the superior aspect of the posterior facet, and the superior aspect of the posterior facet and the anterior calcaneal process. In (B) a postoperative lateral foot radiograph is shown status post open reduction with internal fixation with a 3.5 mm lateral calcaneus plate with an additional two 2.7 mm lag screws underneath the posterior facet. There is restoration of Bohler’s angle measuring 25.2 degrees.

Both cohorts resulted in a satisfactory reduction, as evidenced by acceptable ranges of Bohler’s angle at all time points. Preoperatively, the average Bohler’s angle for the ORIF group was 2.5 degrees (range: −25.0 to 28.1 degrees) and 8.0 degrees for the CRPF group (range: −40.4 to 36.0 degrees). In the immediate postoperative period, the average Bohler’s angle was 33.1 degrees (range 16.2–44.4 degrees) and 30.1 degrees (range 29.4–39.3 degrees) for the ORIF and CRPF groups, respectively. At the final follow-up the average measured Bohler’s angle was 25.4 degrees (range: −5.0 to 41.9 degrees) for the ORIF group and 25.0 degrees (range: −4.6 to 39.2 degrees) for the CRPF group. Examples of pre and postoperative Bohler’s angles for CRPF and ORIF groups can be seen in Figures 1 and 2, respectively.

## Discussion

Despite technical advances in the operative management of intra-articular calcaneus fractures, these injuries remain a challenge to both patients and orthopedic surgeon. As shown in both this study and throughout the literature [2, 7, 10, 17–20], both open and percutaneous techniques result in an adequate reduction of the posterior facet of the calcaneus, yet patients continue to develop significant postoperative complications and morbidity. This study found high rates of both superficial and deep infection, particularly in the ORIF cohort (48%). We also found a high number of reoperations in both cohorts, the majority of which were for removal of calcaneal instrumentation (62%). Post-traumatic subtalar osteoarthritis occurred in 21% of the study population, regardless of maintenance of Bohler’s angle at final follow-up in both cohorts (average 25.4 degrees for ORIF and 25.0 degrees for CRPF).

This study was limited by its retrospective nature, completeness of the EMR, patient compliance and follow-up, and the heterogeneous nature of our two cohorts. This precluded us from directly comparing radiographic outcomes and complications between the two groups. It also prevented accurate analysis of pain scores, pre and postoperative subtalar range of motion, and patient-reported outcome measurements (PROM). However, published metanalysis of calcaneal fractures has shown no difference in pain between open and minimally invasive techniques [23]. Additional complications may have occurred outside the window of follow-up or at outside institutions. We attempted to minimize this bias by including those patients with less than six months follow-up who experienced postoperative complications. Another limitation of this study was the lack of a formal operative algorithm guiding treatment. As previously mentioned, patients with two-part posterior facet fractures within two weeks of injury were generally treated with CRPF, and patients with complex fractures over two weeks were treated with ORIF. Exceptions to this protocol were made in certain patients with high-risk medical comorbidities or social situations. Additionally, the senior author preferred a competence of 20–30 open cases before attempting reduction by closed methods. In doing so, there was a trend of more CRPF cases towards the end of the study, equating to increased operative experience associated with many cases in the CRPF cohort. Finally, this study was limited by the lack of consistent postoperative CT scans to assess the reduction of the posterior facet and axial alignment of the foot and the development of subtalar arthrosis. The senior author did not obtain postoperative CT scans on all patients to control healthcare costs, utilization, and resources. This was a necessary action as this work was done at a large, for-profit, inner-city hospital. If a patient had evidence of delayed union on radiographs or clinical or radiographical evidence of subtalar arthrosis, then a CT scan would be ordered at 6–12 months postoperatively.

A major challenge facing the management of intra-articular calcaneal fractures is the high incidence of postoperative surgical site infections and wound complications [1]. The deep and superficial infection rates in our study were higher than what previous reports indicate [2, 5, 6, 10–13]. This is likely a multifactorial consequence of the patient comorbidities at our institution and the lack of early postoperative follow-up for patients without complications, preventing study inclusion. Although no direct comparisons could be made, the trend of higher infection rates with ORIF remains consistent with prior reports. This may explain the increasing use of minimally invasive techniques in managing intra-articular calcaneal fractures [21]. Conversely, one prior study found no difference in infection rate between open and minimally invasive techniques, especially when the calcaneal artery is patent [4].

A large percentage of our study population needed return trips to the operating room, and the most common reason for this was the removal of instrumentation (62%). Angers-Goulet et al. in 2018 showed a much lower removal of instrumentation rate of 13.6% in their study population at 2 years, with a higher proportion of screw removal in illicit drug users and in patients with inflammatory arthritis [14]. Guo et al. in 2021 similarly found significantly higher rates of implant removal in their own cohort of minimally invasive ORIF procedures compared with percutaneously fixed calcaneus fractures [15].

Disruptions of the posterior facet may lead to the development of post-traumatic subtalar arthritis and a subsequent decrease in patient quality of life [3, 16]. In 2013, Agren et al. conducted a prospective randomized study of 82 patients with displaced intra-articular calcaneal fractures [3]. They reported a significant long-term benefit to operative fixation (open or percutaneous) over conservative treatment in terms of reducing pain, improving functional scores, and decreasing the incidence of post-traumatic subtalar arthritis. We found that 24% of ORIF patients and 36% of CRPF patients experienced symptomatic post-traumatic subtalar arthritis. Our post-CRPF 20% subtalar fusion rate is similar to reported arthrodesis rates of 15% following percutaneous fixation [16].

The immediate postoperative Bohler’s angles in both cohorts fell within acceptable ranges (20–40 degrees), indicating that both operative interventions were successful in restoring calcaneal height. The similar final follow-up Bohler’s angles in each group also suggest that both surgical treatments allow for maintenance of stable fixation. This agrees with current evidence indicating no difference in radiological outcomes between open and minimally invasive procedures [2, 17–20].

Much debate rests on utilizing the traditional extensile lateral approach or minimally invasive techniques [24]. Those in favor of extensile approaches argue improved visualization results in more anatomic reduction, thereby mitigating the likelihood of the development of post-traumatic arthritis [3, 16, 24, 25]. In contrast, a prospective study utilizing postoperative CT-based assessment has shown minimally invasive techniques allow adequate anatomic reduction and stable fixation [7]. Proponents of minimally invasive techniques cite the decreased time from initial injury to surgery, an independent factor shown to significantly reduce infection rates in calcaneus fractures [10], as well as the lower infection rates typically associated with this technique. A full review of these techniques is beyond the scope of this study; however, this information can be found in a 2017 review article published by Dhillon and Prabhakar [25].

## Conclusion

This descriptive study of operative fixation of intra-articular calcaneal fractures utilizing CRPF and ORIF shows both techniques can result in adequate radiographic outcomes with the maintenance of reduction when used in the appropriate patient. CRPF may be the preferred operative treatment in patients at higher risk for infection, such as diabetics and smokers. Also, reoperation is common in these patients and is mostly attributed to the removal of instrumentation. Further prospective randomized controlled trials need to be done to definitively state which operative technique is superior for patients with these difficult injuries.

## Conflicts of interest

The authors have no conflicts of interest to disclose.

## Funding

This research did not receive any specific funding.

## Ethical approval

This study received ethical approval from the Ethics Committee at Detroit Receiving Hospital under protocol number 20-05-2325.

## Informed consent

Written informed consent was obtained from all patients and/or families.

## Authors’ Contributions

Kevin Steelman, MD: Primary author of manuscript. Participated in all aspects of the creation of this manuscript. Nicholas Bolz, MD: Participated in writing and editing the manuscript, as well as data collection. Enrique Feria-Arias, MD: Participated in data collection and manuscript edits. Robert Meehan, MD: Senior author of this study, and performed all surgeries listed in this manuscript.

## References

[1] Mitchell MJ, McKinley JC, Robinson CM (2009) The epidemiology of calcaneal fractures. Foot 19(4), 197–200. 10.1016/j.foot.2009.05.001 20307476

[2] Kline AJ, Anderson RB, Davis WH, Jones CP, Cohen BE (2013) Minimally invasive technique versus an extensile lateral approach for intra-articular calcaneal fractures. Foot Ankle Int 34, 773–780. 23460669 10.1177/1071100713477607

[3] Agren PH, Wretenberg P, Sayed-Noor AS (2013) Operative vs. nonoperative treatment of displaced intra-articular calcaneal fractures: a prospective, randomized, controlled multicenter trial. J Bone Joint Surg Am. 95, 1351–1357. 23925738 10.2106/JBJS.L.00759

[4] Bibbo C, Ehrlich DA, Nguyen HM, Levin LS, Kovach SJ (2014) Low wound complication rates for the lateral extensile approach for calcaneal ORIF when the lateral calcaneal artery is patent. Foot Ankle Int 35, 650–656. 24986898 10.1177/1071100714534654

[5] DeWall M, Henderson CE, McKinley TO, Phelps T, Dolan L, Marsh JL (2010) Percutaneous reduction and fixation of displaced intra-articular calcaneus fractures. J Orthop Trauma 24, 466–472. 20657248 10.1097/BOT.0b013e3181defd74

[6] Abidi NA, Dhawan S, Gruen GS, Vogt MT, Conti SF (1998) Wound-healing risk factors after open reduction and internal fixation of calcaneal fractures. Foot Ankle Int 19, 856–861. 9872474 10.1177/107110079801901211

[7] Nosewicz T, Knupp M, Barg A, et al. (2012) Mini-open sinus tarsi approach with percutaneous screw fixation of displaced calcaneal fractures: a prospective CT-based Study. Foot Ankle Int 33, 925–933. 23131437 10.3113/FAI.2012.0925

[8] Spagnolo R, Bonalumi M, Pace F, Capitani D (2010) Calcaneus fractures, results of the sinus tarsi approach: 4 years of experience. Eur J Orthop Surg Traumatol 20, 37–42.

[9] Tomesen T, Biert J, Frolke JP (2011) Treatment of displaced intra-articular calcaneal fractures with closed reduction and percutaneous screw fixation. J Bone Joint Surg Am 93, 920–928. 21593367 10.2106/JBJS.H.01834

[10] Al-Mudhaffar M (2009) Wound complications following operative fixation of calcaneal fractures. Injury 31, 461–464. 10.1016/s0020-1383(00)00026-7 10831747

[11] Hsu AR, Anderson RB, Cohen BE (2015) Advances in surgical management of intra-articular calcaneus fractures. J Am Acad Orthop Surg 23, 399–407. 26111874 10.5435/JAAOS-D-14-00287

[12] Knapik DM, Hermelin MJ, Tanenbaum JE, Vallier HA (2019) Early complications following articular calcaneus fracture repair: evaluation of open versus percutaneous techniques. J Ortho Trauma Assoc Intl 2, e049. 10.1097/OI9.0000000000000049 PMC7997092 33937677

[13] Sanders R (2000) Displaced intra-articular fractures of the calcaneus. J Bone Joint Surg 82, 225–250. 10682732 10.2106/00004623-200002000-00009

[14] Angers-Goulet M, Beauchamp-Chalifour P, Laflamme N, Bouchard M, Laflamme M (2018) Risk factors for removal of calcaneus screws: a retrospective study. J Foot Ankle Surg 57(4), 701–706. 29703456 10.1053/j.jfas.2017.12.006

[15] Guo C, Xu Y, Li C, Li X, Wang Z, Cai M, Xu X (2021) Comparing less invasive plate fixation versus screw fixation of displaced intra-articular calcaneus fracture via sinus tarsi approach. Int Orthop 45(9), 2231–2237. 33145609 10.1007/s00264-020-04867-5

[16] Schepers T, Schipper IB, Vogels LMM, et al. (2007) Percutaneous treatment of displaced intra-articular calcaneal fractures. J Orthop Sci 12, 22–27. 17260113 10.1007/s00776-006-1076-z PMC2778659

[17] Buckley RE, Tough S (2004) Displaced intra-articular calcaneal fractures. J Am Acad Orthop Surg 12, 172–178. 15161170 10.5435/00124635-200405000-00005

[18] Buckley R, Tough S, McCormack R, et al. (2002) Operative compared with nonoperative treatment of displaced intra-articular calcaneal fractures. J Bone Joint Surg 84, 1733–1744. 12377902 10.2106/00004623-200210000-00001

[19] Xia S, Lu Y, Wang H, Wu Z, Wang Z (2014) ORIF with conventional plate via l-shaped lateral approach vs. internal fixation with percutaneous plate via a sinus tarsi approach for calcaneal fractures: a randomized controlled trial. Int J Surg 12, 475–480. 24607889 10.1016/j.ijsu.2014.03.001

[20] Zhang T, Su Y, Chen W, Zhang Q, Wu Z, Zhang Y (2014) Displaced intra-articular calcaneal fractures treated in a minimally invasive fashion: longitudinal approach vs sinus tarsi approach. J Bone Joint Surg Am 96, 302–309. 24553886 10.2106/JBJS.L.01215

[21] Sanders R (2000) Displaced intra-articular fractures of the calcaneus. J Bone Joint Surg 82, 225–250. 10682732 10.2106/00004623-200002000-00009

[22] Sanders R, Fortin P, Dipasuale T, Walling A (1993) Operative treatment in 120 displaced intra-articular calcaneal fractures: results using a prognostic computed tomography scan classification. Clin Orthop 290, 87–95. 8472475

[23] Gougoulias N, Khanna A, McBride DJ, Maffulli N (2009) Management of calcaneal fractures: systematic review of randomized trials. Br Med Bull 92, 153–167. 19734165 10.1093/bmb/ldp030

[24] Wei N, Zhou Y, Chang W, Zhang Y, Chen W (2017) Displaced intra-articular calcaneal fractures: classification and treatment. Orthopedics 40(6), e921–e929. 29116324 10.3928/01477447-20170907-02

[25] Dhillon MS, Prabhakar S (2017) Treatment of displaced intra-articular calcaneus fractures: a current concepts review. SICOT J 3, 59. 29034875 10.1051/sicotj/2017044 PMC5642053

